# Clinical use of ampicillin-sulbactam/ceftazidime-avibactam combination therapy for carbapenem-resistant *Acinetobacter baumannii* complex infections

**DOI:** 10.1128/aac.00587-26

**Published:** 2026-08-27

**Authors:** Anna Schneider, Kelly A. Cairns, Elke van Gorp, Nenad Macesic, Mariel Beiers, Ben Vezina, Jack Skeggs, Ruzeen Patwa, Matthew Parker, Nhu Quynh Doan, Anton Y. Peleg, Iain J. Abbott, Sadid F. Khan

**Affiliations:** 1 Department of Infectious Diseases, The Alfred Hospital and School of Translational Medicine, Monash University 589641, Melbourne, Victoria, Australia; 2 Microbiology Unit, Department of Pathology, The Alfred Hospital, Bayside Health, Melbourne, Victoria, Australia; 3 Pharmacy Department, The Alfred Hospital, Bayside Health, Melbourne, Victoria, Australia; 4 Centre to Impact AMR, Monash University, Clayton, Victoria, Australia; 5 Infection Program, Department of Microbiology, Monash Biomedicine Discovery Institute, Monash University 214149, Clayton, Victoria, Australia; Houston Methodist Hospital and Weill Cornell Medical College, Houston, Texas, USA

**Keywords:** synergy, *Acinetobacter*, antimicrobial resistance

## LETTER

Sulbactam-durlobactam is a key drug in the treatment of carbapenem-resistant *Acinetobacter baumannii-calcoaceticus* complex (CRAB) infections; however, access is limited due to cost and availability (1, 2). There is increasing *in vitro,* but limited clinical data supporting the utility of avibactam instead of durlobactam in “sulbactam-avibactam” combinations (3–11).

Three patients received ampicillin-sulbactam/ceftazidime-avibactam (AS/CA) for CRAB infections—ventilator-associated pneumonia, infected burns, and bacteremia. Table 1 summarizes clinical features, antimicrobial dosing, and outcomes; case descriptions are provided in the supplemental material. There was no attributable drug toxicity.

**TABLE 1:** Clinical summary data including drug exposure and outcome assessment^*d*^

Case	Syndrome	Comorbidity	Drug dose	Frequency and method^*a*^	Concurrent antibiotic	Renal function (start of Rx)	Renal function (end of Rx)	Weight	Treatment duration	Clinical response (end of Rx)	Microbiological response^*b*^ (day of Rx)	30-day mortality
1	Ventilator-associated pneumonia	Metastatic mesothelioma	AS: 6 g/3 g; CA^*c*^: 2 g/0.5 g	IV q8H over 4 h infusion	Aztreonam 2 g IV q8H over 4 h infusion (until D9)	Cr 159, eGFR 36	Cr 85, eGFR 77	77 kg	17 days	No	Unknown	Deceased
2	Septic shock: infected burns	No	AS: 6 g/3 g; CA^*c*^: 2 g/0.5 g	IV q8H over 2 h infusion	None	Cr 212, eGFR 26	Cr 49, eGFR >90	60 kg	19 days	Yes	Yes (D11, D18)	Alive
3	Septic shock: bacteremia secondary infected burns and CLABSI	Psychosis	AS: 6 g/3 g; CA: 2 g/0.5 g	IV q8H over 2 h infusion	TMP/SMX 320 mg/1,600 mg IV q12H	Cr 45, eGFR >90	Cr 45, eGFR >90	59 kg	10 days	Yes	Yes (D3, D6, D10)	Alive

^*a*^ Both drugs were co-administered simultaneously through separate lumens of a central line.

^*b*^ Microbiological response defined by negative culture from initial culture-positive site. Day of Rx refers to which day of treatment the follow-up culture was performed—see the supplemental material.

^*c*^ Initial CA dose reduction for acute kidney injury—see the supplemental material.

^*d*^ CLABSI, central line-associated bloodstream infection; AS, ampicillin/sulbactam; CA, ceftazidime/avibactam; Rx, treatment; Cr, creatinine (µmol/L); eGFR, estimated glomerular filtration rate (mL/min); TMP/SMX, trimethoprim/sulfamethoxazole.

Isolates underwent whole-genome sequencing with Oxford Nanopore (supplemental material and Table S2). All isolates carried acquired *bla*_OXA-23_, with IS*Aba1* detected as the upstream promoter for intrinsic *bla*_OXA-51-like_ and *bla*_ADC_ genes. Additionally, a PBP3 mutation (A515V) associated with increased sulbactam-durlobactam MICs was present in all isolates (12).

Broth microdilution MIC measurements were performed for all components for AS/CA (Table 2 and supplemental material). The median sulbactam MIC of 32 mg/L was reduced to a median of 8 mg/L with the addition of avibactam. Synergy checkerboard assays were performed to calculate the fractional inhibitory concentration index (FICi). *F*_min_ is the lowest determined FICi value, and *F*_min_ ≤ 0.5 indicates synergy (Table 3 and supplemental material) (13). The sulbactam-avibactam combination showed synergy for all isolates (*F*_min_, 0.188–0.125). Testing of ampicillin-sulbactam with ceftazidime-avibactam when sulbactam and avibactam concentrations were both fixed at 2 mg/L and 4 mg/L did not demonstrate further synergy from the partner drugs (*F*_min_, 1.001–0.501), but some additive benefit was observed.

**TABLE 2:** Broth microdilution MIC measurements of tested antimicrobials and combinations^*c*^

Antimicrobial	MIC (mg/L, range)		
	Case 1	Case 2	Case 3
Ampicillin^*b*^	>256	>256	>256
Ceftazidime^*b*^	>256 (256 to >256)	>256	>256
Sulbactam^*b*^	32	32	32 (32–64)
Avibactam	>64	>64	>64
Ampicillin-sulbactam^*a*^ (sulbactam fixed 4 mg/L)	>256/4	>256/4	>256/4
Ampicillin-sulbactam^*a*^ (2:1 ratio)	64/32	64/32	64/32
Ceftazidime-avibactam^*a*^ (avibactam fixed 4 mg/L)	128/4	64/4 (64/4 to 128/4)	64/4
Ceftazidime-avibactam (2:1 ratio)	64/32	32/16 (32/16 to 64/32)	32/16
Sulbactam-avibactam (avibactam fixed 4 mg/L)	16/4 (8/4 to 16/4)	8/4 (4/4 to 8/4)	8/4 (4/4 to 8/4)
Ampicillin-sulbactam-avibactam^*b*^ (sulbactam and avibactam fixed 2 mg/L)	>256/2/2	>256/2/2	>256/2/2
Ampicillin-sulbactam-avibactam^*b*^ (sulbactam and avibactam fixed 4 mg/L)	>256/4/4	>256/4/4	>256/4/4
Ceftazidime-avibactam-sulbactam^*b*^ (sulbactam and avibactam fixed 2 mg/L)	128/2/2	64/2/2	64/2/2
Ceftazidime-avibactam-sulbactam^*b*^ (sulbactam and avibactam fixed 4 mg/L)	64/4/4	32/4/4	32/4/4

^*a*^ ATCC reference strains (ATCC 25922, ATCC 35218, ATCC 700603, ATCC 27853, and ATCC 29213) were tested in parallel and in triplicate, and measurements were within EUCAST and CLSI ranges, where applicable.

^*b*^ Tested as a single replicate.

^*c*^ Unless indicated, BMD MIC measurements were tested in triplicate, the median MIC value is presented with the range in parentheses, where necessary.

**TABLE 3:** Checkerboard synergy assays

Checkerboard antimicrobial combination	Checkboard synergy (*F*_min_)^*a*^		
	Case 1	Case 2	Case 3
Sulbactam + avibactam	0.188	0.125	0.188
Ampicillin-sulbactam + ceftazidime-avibactam (sulbactam and avibactam fixed 2 mg/L)	1.001	0.501	0.501
Ampicillin-sulbactam + ceftazidime-avibactam (sulbactam and avibactam fixed 4 mg/L)	1.001	0.501	0.501

^*a*^ The FICi was calculated for wells without growth (≤10% relative OD compared to the growth control well) adjacent to wells with growth (>10% relative OD). FIC = (*C*_*A*_/MIC_*A*_) + (*C*_*B*_/MIC_*B*_), where *A* is the concentration of one drug, and *B* is the concentration of the other drug in each well. The *F*_min_ is defined as the lowest FIC-value and the *F*_max_ as the highest FIC-value. If, *F*_max_ > 2, the combination is defined as antagonistic, regardless of *F*_min_. If *F*_min_ ≤ 0.5, the combination is defined as synergistic, when each drug’s MIC drops by at least two dilution steps when combined. If *F*_min_ values are 0.5–2, the combination is considered to have indifference or some additional activity. ATCC 25922 was included as a control strain in each checkerboard set-up. *F*_min_ values were 0.75–1.50.

We demonstrate potential clinical efficacy and tolerability of AS/CA for the treatment of serious CRAB infections. Case 1 did not clearly demonstrate clinical response, and the cause of death was progressive metastatic mesothelioma. Microbiological response could not be evaluated due to lack of follow-up cultures. Cases 2 and 3 had no growth on burn cultures while on treatment, despite having been consistently culture-positive before treatment. With discontinuation of AS/CA, both cases re-isolated CRAB from burn samples, though neither required re-treatment for clinical infection.

The contribution of concomitant antimicrobials in our cases was likely limited. In Case 1, initial therapy included aztreonam for possible concurrent infection with MBL-producing Enterobacterales. Case 3 developed bacteremia while receiving trimethoprim-sulfamethoxazole (to which it tested susceptible), resulting in the addition of AS/CA, suggesting that trimethoprim-sulfamethoxazole alone was insufficient.

Notably, all three isolates contained a PBP3 mutation that has been associated with higher sulbactam/avibactam MICs (8). Findings in our isolates are similar (MICs 8/4 to 16/4 mg/L), but did not appear to result in treatment failure. The sulbactam dose in standard dosing of sulbactam-durlobactam (1 g/1 g IV every 6 h over 3 h infusion) is 4 g/day (1, 2), contrasted against the total sulbactam dose of 9 g/day in all three cases. The very high exposures of sulbactam and optimization of drug administration may have provided efficacy at these elevated MICs.

In conclusion, these data provide a foundation for further study and best clinical utilization of AS/CA for serious CRAB infections. This is of critical importance, as effective and non-toxic options for the treatment of CRAB are limited. Sulbactam-durlobactam therapy is inaccessible in areas where need is dire, and in such scenarios, AS/CA may serve as an appropriate alternative.

## Data Availability

Whole-genome sequencing data are publicly available under NCBI GenBank BioProject accession number PRJNA1507525.

## SUPPLEMENTAL MATERIAL

Supplementary material aac.00587-26-s0001.docx Case descriptions, antimicrobial administration details; Supplemental methods; Table S1.

Table S2 aac.00587-26-s0002.xlsx Whole genome sequencing data.

ASM does not own the copyrights to Supplemental Material that may be linked to, or accessed through, an article. The authors have granted ASM a non-exclusive, world-wide license to publish the Supplemental Material files. Please contact the corresponding author directly for reuse.
