# Supplementary material for: Clinical use of ampicillin-sulbactam/ceftazidime-avibactam combination therapy for carbapenem-resistant Acinetobacter baumannii complex infections
Source: Antimicrob Agents Chemother. 2026 Aug 27;70(10):e00587-26. doi: 10.1128/aac.00587-26 (PMC13689153; doi:10.1128/aac.00587-26)
Supplement: Supplementary file 1 [file aac.00587-26-s0001.docx]

**SUPPLEMENTARY MATERIAL**

**CASE DESCRIPTIONS**

**Case 1**

A 75-year-old man with mesothelioma was repatriated to our intensive care unit (ICU) following a 3-week ICU admission in Indonesia after a multi-trauma motor vehicle accident. In the week prior to transfer, the patient had started treatment for a ventilator associated pneumonia (VAP) due to carbapenem resistant *Klebsiella pneumoniae* and *Enterobacter cloacae* complex with a combination of meropenem, colistin, and cefiderocol, complicated by acute kidney injury (Cr 318 µmol/L; eGFR 16 mL/min).

CT chest imaging upon arrival demonstrated pulmonary infiltrates, complex bilateral pleural effusions and bilateral rib fractures, with associated hypoxic respiratory failure. Purulent endotracheal secretions and bronchoalveolar lavage (BAL) samples grew *bla*_OXA-48_ producing *K. aerogenes* and *bla*_OXA-23_ producing carbanepem-resistant *Acinetobacter baumannii-calcoaceticus* complex (CRAB) (Supplementary Table 1 for full AST). Rectal screening swabs demonstrated *bla*_NDM-4_ producing *K. pneumoniae* and *bla*_KPC_ producing *Pseudomonas aeruginosa*, but these were never isolated from clinical samples.

The patient was initially treated with ceftazidime-avibactam and aztreonam targeting the carbapenem resistant Enterobacterales. Determining whether the CRAB was colonising the respiratory tract or causing a ventilator associated pneumonia was challenging. Due to limited clinical improvement and ongoing isolation of CRAB from further BAL samples treatment was modified. On day 20 of admission, ampicillin-sulbactam 6g/3g IV 8-hourly was added to ceftazidime-avibactam 1g/0.25g IV 8-hourly, administered simultaneously over a 4-hour infusion for treatment of possible VAP without features of sepsis or shock. The ceftazidime-avibactam dose was increased to 2g/0.5g on day 7 of treatment as renal function improved (Cr 107 µmol/L; eGFR 58 mL/min). Aztreonam continued empirically until day 9 of AS/CA therapy.

There was no significant improvement in respiratory failure. The patient underwent pleural decortication which revealed grossly abnormal lung and pleura that was consistent with progressive mesothelioma, and tissue cultures were negative. A PET scan was suggestive of metastatic mesothelioma. Consequently, the patient’s goals of care were revised, and the patient transitioned to end of life care and died. Microbiological response to therapy could not be assessed due to lack of follow up respiratory cultures. There were no attributable adverse effects to AS/CA and treatment continued for 17 days until transition of care.

**Case 2**

A 34-year-old woman was repatriated to our ICU following a 3-day overseas ICU admission in Indonesia due to a fuel explosion resulting in 50% total body surface area (TBSA) burns. Burns and endotracheal aspirate cultures grew *bla*_OXA-23_ producing CRAB (Supplementary Table 1 for full AST) and a wild type *P. aeruginosa*.

Day 4 of admission the patient developed fevers with new vasopressor requirement and acute kidney injury (Cr 212 µmol/L; eGFR 26 mL/min). The burns had significant bleeding with clinical concern for infection. There were no features of VAP. The patient was commenced on ampicillin-sulbactam 6g/3g IV 8-hourly and ceftazidime-avibactam 1g/0.25g IV 8-hourly, administered simultaneously over a 2-hour infusion. Blood cultures were not collected prior to administration of empiric antimicrobials, limiting exclusion of bloodstream infection. The ceftazidime-avibactam dose was increased to 2g/0.5g on day 3 of treatment with improvement to baseline renal function.

The patient improved, with cessation of vasopressors, resolution of acute kidney injury, and defervescence by day 3 of treatment. Treatment continued for 19 days, during which burns cultures on day 11 and day 18 of therapy became culture negative for CRAB. The patient developed new burns infection on day 18 of therapy, with first isolation of a *bla*_IMP-7_ producing requiring change in antimicrobial regimen. There were no attributable adverse effects to AS/CA treatment.

The patient was discharged after a 3-month inpatient admission, during which she required further treatment for *P. aeruginosa*. Burns swabs became recolonised with CRAB 13 days after cessation of AS/CA but did not require retreatment for CRAB infection.

**Case 3**

A 43-year-old man with psychosis was admitted after self-immolation with 55% TBSA burns. On day 6 of admission the patient underwent debridement of clinically infected burns without features of sepsis or shock, cultures from which grew *bla*_OXA-23_ producing CRAB (Supplementary Table 1 for full AST). Endotracheal aspirate and BAL cultures also isolated CRAB, without features of VAP. The patient was commenced on trimethoprim-sulfamethoxazole at 320mg/1600mg IV 12-hourly (10mg/kg/day trimethoprim component), for which the isolate tested susceptible.

On day 13 the patient developed septic shock with rising vasopressor requirements. Ampicillin-sulbactam 6g/3g IV 8-hourly and ceftazidime-avibactam 2g/0.5g IV 8-hourly were added, administered simultaneously over a 2-hour infusion. Blood cultures from a central line grew CRAB, and the central line was subsequently removed. There was defervescence and cessation of vasopressors by day 3 of treatment, improvement of infected burns, with cessation of all antimicrobials at day 10. Repeat blood cultures on day 3 of treatment were negative. During the period of treatment burns cultures became negative for CRAB (day 6 and day 10 of treatment), however the patient became recolonised 3 days after cessation of AS/CA. There were no attributable adverse effects to AS/CA treatment.

The patient was discharged to rehabilitation after 4-months and did not require re-treatment with AS/CA for invasive CRAB infection.

**DRUG ADMINISTRATION**

Antimicrobials were administered concurrently as prolonged infusions to optimise antimicrobial exposure. All patients had multi-lumen central lines in-situ. Whilst Case 2 and 3 received concurrent prolonged infusions of AS/CA over 2-hours, extended infusions over a minimum of 4-hours (Case 1) may be considered, especially in severe infections. Continuous infusions over 24 hours would provide the greatest time above MIC for optimised exposure, however drug stability is a limitation. Continuous infusions of ampicillin/sulbactam 27g (18g ampicillin, 9g sulbactam) can be utilised [1], however current evidence suggests ceftazidime/avibactam should be administered as two 12-hour continuous infusions [2,3]. There is an absence of data regarding compatibility of co-infusing ampicillin-sulbactam with ceftazidime-avibactam, and we therefore opted to infuse these drugs through separate lumens of the central line.

**EVALUATION FOR ANTIMICROBIAL TOXICITY**

As determined by judgement of the clinicians at time of treatment, no patient required cessation of any of components AS/CA due to tolerability or toxicity. Additional retrospective case review of the electronic medical record was conducted evaluating for potential neurotoxicity, nephrotoxicity, hepatotoxicity, cytopenias, diarrhoea (including *Clostridiodes difficile*), and allergic drug reactions – of which no evidence was found. All patients were intubated and sedated for their duration of therapy in intensive care, and assessments around subjective symptoms (e.g. nausea) could not be determined.

**EVALUATION OF MICROBIOLOGICAL RESPONSE**

With reference to Table 1 in the main manuscript, a successful microbiological response was defined by a subsequent negative culture from an initially culture positive site. “Day of Rx” refers to which day of treatment the follow up culture was performed. e.g. A positive blood culture at diagnosis with negative follow up blood cultures on day 3 of treatment would be defined as a successful microbiological response (D3). Due to the nature of retrospective case evaluation, the availability and timing of follow up cultures occurred at the judgement of clinicians through the routine provision of clinical care.

**SUPPLEMENTARY METHODS**

**Whole genome sequencing and bioinformatic analysis**

All bacterial isolates were cultured in heart infusion (HI) broth (Thermo Fisher Scientific) for 18 hours at 37°C, shaking at 200 rpm. Genomic DNA was extracted using the GenFind V3 Reagent Kit (Beckman Coulter) as per manufacturer’s instructions. Long-read sequencing was prepared using the Rapid Barcoding Kit V14 (Oxford Nanopore Technologies) and sequenced on the MinION instrument with an R10.4.1 flow cell (Oxford Nanopore Technologies) for 48 hours. Basecalling was performed with Dorado v0.5.1 using the ‘super’ basecalling model. Reads were filtered using Filtlong v.0.2.0 with the following parameters: ‘--min_length 1000 --keep_percent 90 --target_bases 500000000’. The reads have been deposited in the NCBI Sequence Read Archive under project accession PRJNA1507525.

Assemblies were created using Hybracter v.0.11.0 with standard parameters. Assembly quality was checked using Quast v5.2.0. Assemblies were annotated using bakta v1.11.4. We determined *in silico* multi-locus sequence type (ST) using mlst v.2.32.2. AMRFinderPlus v3.12.8 was used for antimicrobial resistance detection, using default settings and the NCBI curated database. Assemblies were visually inspected for presence of IS*Aba1* in promoter regions of *bla*_OXA-23,_ *bla*_OXA-51_-like and *bla*_ADC_ genes. The PBP1a (*ponA*), PBP1b (*mcrB*), PBP2 (*mrdA*) and PBP3 (*ftsI*) were evaluated for the presence of mutations. Pairwise single nucleotide polymorphism differences were estimated using kbo v0.5.1.

Isolates from all three cases were identified as sequence type 2 (Pasteur scheme). Isolates from case 2 and 3 were part of a clonal outbreak (4 single nucleotide polymorphism [SNP] differences). The isolate from case 1 had 325 SNPs compared to the other two isolates, indicating a distinct strain.

**Antimicrobial susceptibility testing and synergy checkerboard assays**

(i) *Antibiotics*

Ampicillin (Merck Life Science PTY LTD, A9393), sulbactam (Merck Life Science PTY LTD, PHR2576) and ceftazidime (Merck Life Science PTY LTD, PHR1847) were prepared to a stock solution of 10000 mg/L. Ampicillin was dissolved in sterile phosphate buffer, pH 6.0, 0.1 mol/L. Sulbactam was dissolved in sterile water. Ceftazidime was dissolved in anhydrous sodium carbonate at a 10% w/w relative to ceftazidime, and further diluted in sterile water. Avibactam (Novachem superior standards, CS-4112) was prepared as a 5000 mg/L stock solution in sterile water. All stock solutions were filtered, aliquoted and stored at -80°C until use. Subsequent dilutions were prepared in Mueller-Hinton II (MH-II) broth (Becton Dickinson).

(ii) *Broth microdilution MIC testing*

MICs for all antibiotics were determined, in triplicate unless indicated, according to ISO broth microdilution standard methodology. Antibiotic concentration ranges were 0.25 to 256 mg/L for all antibiotics except avibactam (0.06 to 64 mg/L). Microdilution plates were incubated at 35°C for 16-20 hours. ATCC 25922 (*E. coli*), ATCC 35218 (*E. coli*), ATCC 700603 (*K. pneumoniae*), ATCC 27853 (*P. aeruginosa*) and ATCC 29213 (*S. aureus*) were used as quality control isolates.

(iii) *Synergy checkerboard assays*

The combinations sulbactam plus avibactam, and ampicillin-sulbactam plus ceftazidime-avibactam were further studied in a 12x8 checkerboard assay as described by Berkhout *et al.* [4]. 25 µL of both drugs were added to each well. Final drug concentrations for the sulbactam plus avibactam checkerboard ranged from 0.06 to 64 mg/L (sulbactam) and from 1 to 64 mg/L (avibactam). The ampicillin-sulbactam plus ceftazidime-avibactam checkerboard was performed with fixed concentrations of avibactam and sulbactam, both tested at 2 and 4 mg/L. The final concentrations of ampicillin ranged from 0.25 to 256 mg/L and for ceftazidime from 2 to 128 mg/L. Plates were incubated at 35°C for 16-20 hours. Optical densities (OD) of each well were measured with the Tecan i-control plate reader at OD=600 nm. ODs were converted to relative OD%, compared with the growth control after subtracting background OD. Analysis was performed using the Fractional Inhibitory Concentration index (FICi) as described by Meletiadis et al [5]. F_min_ was defined as the lowest FICi-value, and F_max_ was defined as the highest FICi-value. An F_min_ ≤ 0.5 indicates synergy, while an F_max_ > 2 indicates antagonism.

**SUPPLEMENTARY TABLES**

**Table S1: Antimicrobial susceptibility testing results for *Acinetobacter baumannii* complex isolates from cases.**

|  | **Case 1** | **Case 2** | **Case 3** |
| --- | --- | --- | --- |
| **Meropenem** | >16 mg/L; R | >16 mg/L; R | >16 mg/L; R |
| **TMP/SMX^a^** | >4 mg/L; R | 1 mg/L; S | 1 mg/L; S |
| **Ciprofloxacin** | >2 mg/L; R | >2 mg/L; R | >2 mg/L; R |
| **Gentamicin** | >8 mg/L; R | >8 mg/L; R | >8 mg/L; R |
| **Tobramycin** | >8 mg/L; R | >8 mg/L; R | >8 mg/L; R |
| **Amikacin** | >32 mg/L; R | >32 mg/L; R | >32 mg/L; R |
| **Colistin** | ≤ 0.5 mg/L; S | ≤ 0.5 mg/L; S | ≤ 0.5 mg/L; S |
| **Cefiderocol** | 24mm; WT | 25mm; WT | 23mm; WT |
| **Tigecycline** | 4 mg/L | 4 mg/L | 4 mg/L |
| **Minocycline** | NP | 8 mg/L | 8 mg/L |

Antimicrobial susceptibility testing was performed by broth microdilution (Thermofisher, Sensititre™, GN6F & EUMDROXF), except for tigecycline and minocycline which were assessed using gradient strip diffusion (BioMérieux), and cefiderocol that was assessed by disc diffusion (Oxoid) and EUCAST interpretations applied. EUCAST methodology used for categorical interpretation where breakpoints or ecological cut-off values are available. NP, not performed; WT, wild type; TMP/SMX, trimethoprim/sulfamethoxazole. ^a^, Concentration expressed as trimethoprim component.

**SUPPLEMENTARY REFERENCES:**

1. Tamma PD, Aitken SL, Anderson DJ, et al. Infectious Diseases Society of America (IDSA) guidance on the treatment of antimicrobial-resistant gram-negative infections. Infectious Diseases Society of America, 2024. Available at: <https://www.idsociety.org/practice-guideline/amr-guidance/>. Accessed 28 January 2026.
2. Naicker S, Roberts JA, Won H, et al. Evaluation of the stability of ceftazidime/avibactam in elastomeric infusion devices used for outpatient parenteral antimicrobial therapy utilising a national stability protocol framework. JAC Antimicrob Resist. 2024 ;6(2) :dlae056
3. Loeuille G, D’Huart E, Vigneron J, et al. Stability studies of 16 antibiotics for continuous infusion in intensive care units and for performing outpatient parenteral antimicrobial therapy. Antibiotics (Basel). 2022 ;11(4) :458.
4. Berkhout J, Melchers MJ, van Mil AC, et al. In vitro activity of ceftazidime-avibactam combination in vitro checkerboard assays. AAC. 2015;59(2): <https://doi.org/10.1128/aac.04146-14>
5. Meletiadis J, Pournaras S, Roilides E, et al. Defining fractional inhibition concentration index cutoffs for additive interactions based on self-drug additive combinations, monte-carlo simulation analysis, and in vitro-in vivo correlation data for antifungal drug combinations against Aspergillus fumigatus. AAC. 2010;54(2): [doi.org/10.1128/aac.00999-09](https://doi.org/10.1128/aac.00999-09)
